# Measurement Invariance and Psychometric Properties of the Self-Concealment Scale in Iran

**DOI:** 10.3390/healthcare14152262

**Published:** 2026-07-24

**Authors:** Mohammad Niroumand Sarvandani, Xue Luo, Hamid Sharifi, Somayeh Naghavi, Shujuan Chen, Qing-Wei Chen

**Affiliations:** 1 HIV/STI Surveillance Research Center, and WHO Collaborating Center for HIV Surveillance, Institute for Futures Studies in Health, Kerman University of Medical Sciences, Kerman 7616913555, Iran; m.niroumand@live.com (M.N.S.); sharifihami@gmail.com (H.S.); 2 Department of Mental Health Education, Guangdong Mechanical & Electrical Polytechnic, Guangzhou 510515, China; nfyy8866@i.smu.edu.cn; 3 Institute for Global Health Sciences, University of California, San Francisco, CA 94143, USA; 4 Department of Psychology, Science and Research Branch, Islamic Azad University, Tehran 1477893855, Iran; saranaqavi@gmail.com; 5 Department of Psychology, School of Education, Ningxia University, Yinchuan 750021, China; chen_sj@nxu.edu.cn; 6 Lab of Light and Physio-Psychological Health, National Center for International Research on Green Optoelectronics, South China Normal University, Guangzhou 510006, China; 7 Guangdong Provincial Key Laboratory of Optical Information Materials and Technology, Institute of Electronic Paper Displays, South China Academy of Advanced Optoelectronics, South China Normal University, Guangzhou 510006, China; 8 School of Psychology, South China Normal University, Guangzhou 510631, China

**Keywords:** self-concealment, psychometrics, reproducibility of results, factor analysis, sex factors, surveys and questionnaires

## Abstract

**Background/Objectives:** Self-concealment is associated with greater psychological distress and decreased help-seeking engagement. This study aimed to evaluate the psychometric properties and sex invariance of the Persian Self-Concealment Scale (SCS) in Iranian adults. **Methods:** Participants were recruited using convenience sampling and social media advertising, targeting adults aged 18 or older who could read and understand Persian. The valid sample comprised 644 adults. Exploratory factor analysis was performed to delineate the underlying structural dimensions, followed by confirmatory factor analysis (CFA). Multi-group CFA was used to evaluate the gender invariance. **Results:** A one-factor exploratory solution explained 48.83% of the variance, whereas parallel analysis suggested three factors, indicating some residual multidimensionality. The initial one-factor CFA showed poor fit (RMSEA = 0.114, CFI = 0.899, TLI = 0.871). A revised one-factor model that accounted for four localized item-pair residual associations showed improved fit in Sample 2 (RMSEA = 0.062, CFI = 0.974, TLI = 0.962, N = 322) and acceptable fit when applied without further respecification to the non-overlapping Sample 1 (N = 322) internal holdout (RMSEA = 0.078, CFI = 0.960). Internal consistency was high (α = 0.896; ω = 0.897), while correlations with depression, anxiety, and stress ranged from 0.35 to 0.37, providing moderate evidence of convergent validity. Gender invariance was partially supported. **Conclusions:** The Persian SCS shows promising but qualified psychometric performance for research use, pending independent replication and longitudinal validation.

## 1. Introduction

Every individual harbors at least one untold secret within themselves, which makes keeping secrets an everyday routine [1,2]. Not only do the subjects and contents of individuals’ secrets vary from person to person, but substantial individual differences also exist in their inclination to conceal or disclose such information [3,4]. Self-concealment is one critical type of those individual differences, which is defined as the inherent predisposition to actively withhold from others personal information that one appraises as distressing or negatively valenced [5].

Self-concealment has received substantial scholarly attention because it has been associated with physical health problems [5,6], interpersonal difficulties [7,8], and psychological maladjustment [9]. The working model of self-concealment [6] proposes that withholding distressing personal information may interfere with support seeking and emotional regulation. Consistent with this account, previous studies have reported positive associations between self-concealment and depression [10], anxiety [11], stress [12], and posttraumatic stress symptoms [13,14,15]. Self-concealment has also been associated with non-suicidal self-injury [16,17,18], suicidality [18,19,20,21,22], alcohol-related problems [22,23], substance misuse [24], disordered eating [25,26,27], and several problematic digital behaviors [16,18,28,29,30].

The Self-Concealment Scale (SCS) is the most widely used instrument for assessing self-concealment [6]. The original English-language SCS [5] has been translated and used in German [31], Japanese [32], Chinese [33], Turkish [34], Portuguese [35], and Spanish [27,36,37] language contexts. Across these settings, internal consistency estimates have generally been acceptable: an integrative review of 99 studies reported a mean coefficient alpha of 0.87 [6], and individual validation studies reported alpha coefficients of 0.80-0.866 [27,31,32,33,35,36] and omega of 0.85 [37]. Evidence for other forms of validity has varied across studies and remains context dependent.

The dimensional structure of the SCS remains unsettled. Most validations of the original 10-item scale have reported a one-factor structure [5,27,32,33,38,39,40,41]. After deleting two items (item 3 and item 10), Salinas-Onate et al. [37] confirmed the one-factor structure as well. Costa et al. [35], however, reported two factors for an expanded version that added two health-related items. Because the present study administered the original 10 items, the one-factor model was evaluated as the primary structural hypothesis; the published expanded two-factor specification could not be reproduced directly without its two additional items.

Measurement invariance is necessary to determine whether a scale measures the same construct across groups [42]. Without adequate invariance, observed group differences may reflect measurement artifacts rather than differences in self-concealment. Previous SCS studies have reported mixed gender-invariance findings [37,40] and limited evidence regarding age invariance [39]. Gender invariance therefore required direct evaluation in the present Iranian sample before interpreting gender comparisons or considering applied use of observed scores.

No culturally validated Persian SCS has been available for use in Iran. The cross-cultural translation and psychometric validation of the SCS into Persian is important because self-concealment may be shaped by sociocultural norms surrounding privacy, family reputation, stigma, and help-seeking. In Iranian contexts, concerns about family honor, social judgment, and stigma surrounding psychological difficulties may influence whether distressing personal information is disclosed or concealed [43,44,45,46,47]. Gendered social expectations may also affect concealment experiences among women and other groups whose private experiences can carry heightened social consequences [44,48]. Clinically, stigma related to psychological suffering can delay or discourage help-seeking, making self-concealment relevant to mental health assessment and intervention in Iran [46,47]. Because the original SCS was developed in Western samples, direct translation alone cannot ensure semantic, conceptual, and measurement equivalence in Iranian adults. Rigorous translation, back-translation, and local psychometric testing are therefore needed to evaluate whether the Persian SCS provides a culturally appropriate and psychometrically defensible measure for Iranian research settings.

Building on existing empirical findings, the current investigation sought to translate the SCS into Persian and evaluate its psychometric properties among Iranian adults, with a specific focus on testing measurement invariance across gender groups. We hypothesized that the Persian-adapted SCS would exhibit a robust unidimensional latent structure and satisfy criteria for gender invariance, thereby functioning as a reliable and valid instrument for assessing self-concealment in this population.

## 2. Materials and Methods

### 2.1. Procedure and Participants

Participants were recruited using a convenience sampling strategy through an online survey from April to December 2024 hosted on DigiSurvey, an Iranian web-based platform designed for electronic data collection. Prior to dissemination, the questionnaire was carefully designed and programmed to ensure clarity, usability, and smooth navigation for respondents. Particular attention was given to the organization of the survey pages, wording of instructions, and technical functionality in order to facilitate completion and reduce response errors.

The survey link was distributed through a standardized invitation message across several widely used social networking and messaging applications, including Telegram, WhatsApp, and Instagram, as well as popular Iranian domestic platforms such as Eitaa, Bale, and Rubika. The invitation message was shared in multiple public and private groups/channels involving users from different cities and regions of Iran, with the aim of reaching a broad adult population and increasing geographic diversity within the sample. The message contained a brief explanation of the study objectives, the voluntary nature of participation, and an invitation to take part in the research.

When participants clicked on the survey link, they were first directed to an electronic informed consent page that explained the purpose of the study, the anonymous and voluntary nature of participation, the confidentiality of responses, and the right to discontinue participation at any point prior to submission. Only individuals who explicitly indicated their consent were allowed to proceed to the questionnaire. Those who did not provide consent could not enter the survey.

To enhance data quality and reduce missing values, all questionnaire items were programmed as mandatory response fields. Thus, participants were required to select one response option for each item before advancing to the next page of the survey. This procedure helped ensure dataset completeness and minimized the occurrence of item-level missing data.

The inclusion criteria were: (1) being 18 years of age or older, (2) being Iranian, and (3) being able to read and understand Persian. Because the study was conducted through an online self-report survey, participants were also required to provide informed consent before completing the questionnaire. Responses were excluded if the questionnaire was not completed or if the submitted data contained substantial missing information.

The final 644 valid sample comprised 429 women (66.6%) and 215 men (33.4%). Age was collected in six categorical brackets: 18–20 (16.0%), 21–30 (37.7%), 31–40 (27.0%), 41–50 (14.4%), 51–60 (4.0%), and 60+ (0.8%); because age was not recorded as a continuous variable, a mean/SD age cannot be reported. Marital status was 46.0% single, 49.5% married, and 4.2% divorced (0.3% missing). Educational attainment was concentrated at the Bachelor’s degree level (34.9%), followed by high school (26.2%) and Master’s degree (20.0%), with smaller proportions at other levels. Most participants were single (46.0%) or married (49.5%). Employment status was distributed across studying (24.1%), full-time employment (23.3%), part-time employment (14.8%), unemployed (14.6%), other (13.5%), freelance (5.7%), and retired (4.0%) (see Table 1 for more details).

### 2.2. Measurements

#### 2.2.1. SCS

The SCS is a 10-item measure that has commonly been modeled as unidimensional [5], exemplified by the item “I have a secret that is so private I would lie if anyone asked me about it”. Each item is scored from 1 (strongly disagree) to 5 (strongly agree). No items are reverse-scored, and the 10 item scores are summed to produce a possible total score of 10–50; higher scores indicate a greater tendency to conceal negative or distressing thoughts and feelings.

The Persian translation of the SCS was developed using a standard forward–backward translation procedure. First, two independent bilingual translators whose native language was Persian separately translated the original English items into Persian. These forward translations were then compared, discussed, and synthesized into a single preliminary Persian version through consensus. The synthesized version was back-translated into English by an independent bilingual translator who was blinded to the original instrument, and the back-translated version was compared with the original scale to ensure conceptual and semantic consistency.

The pre-final Persian version was then reviewed by an expert panel comprising four faculty members from different universities and disciplines, including a counseling psychologist, an educational psychologist, an epidemiologist, and a medical sciences specialist. The panel evaluated the items for semantic equivalence, conceptual consistency, clarity of wording, readability, and cultural appropriateness for use with Iranian adults. The expert panel did not identify any item that required major conceptual modification or substantial cultural adaptation. However, the panel recommended several minor linguistic refinements to improve the fluency, readability, and idiomatic naturalness of the Persian wording while preserving the original meaning of each item. All revisions were made through consensus discussion.

The pre-final Persian version was subsequently pilot-tested with eight adults from the target population to provide a preliminary qualitative assessment of clarity, comprehensibility, readability, and acceptability of the translated items. In order to enhance the usefulness of this pilot phase for detecting potential wording difficulties, the eight participants were purposefully selected to represent diversity in socioeconomic status and educational level. This sampling strategy was used because individuals from different educational and socioeconomic backgrounds may vary in their familiarity with formal or psychological vocabulary, which could affect the comprehensibility of items related to secrecy, emotional distress, and interpersonal disclosure. By including participants with a range of educational and socioeconomic characteristics, we aimed to maximize the likelihood of identifying items that might be difficult to understand or culturally awkward for certain groups.

Feedback from the pilot test indicated that the items were generally understandable and culturally acceptable. No item was judged to be incomprehensible, conceptually ambiguous, or culturally inappropriate. The comments received mainly resulted in minor wording refinements aimed at improving the naturalness and smoothness of the Persian expressions. For example, some revisions focused on selecting more fluent Persian equivalents for idiomatic expressions such as “keep it to myself”, “tormented me”, and “backfires” in order to improve both comprehensibility and psychological relevance in Persian. These changes were linguistic rather than conceptual; no item was removed, replaced, or substantially altered, and the final Persian version remained conceptually consistent with the original English scale.

#### 2.2.2. Patient Health Questionnaire-2 (PHQ-2)

The PHQ-2 [49] contains two items and provides a brief assessment of depressive symptoms [50]. Each item is scored on a 4-point scale from 0 (not at all) to 3 (nearly every day). A Persian translation has shown satisfactory psychometric properties in Iranian samples [51].

#### 2.2.3. Generalized Anxiety Disorder 2-Item Scale (GAD-2)

The GAD-2 [52] consists of two items assessing anxiety symptoms [53]. Each item is scored on a 4-point scale from 0 (never) to 3 (almost every day). A Persian version has been evaluated in Iran [54] and was used in the current study.

#### 2.2.4. Perceived Stress Scale-4 (PSS-4)

The PSS-4 is a four-item measure of perceived stress [55]. Each item is scored on a 5-point scale from 1 (never) to 5 (always). The Iranian version of the PSS-4 [56] was used in this study.

### 2.3. Statistical Analysis

Data analysis was conducted in R. The data were randomly divided into Sample 1 (N = 322; item analysis and Exploratory Factor Analysis (EFA)) and Sample 2 (N = 322; primary CFA, correlations with theoretically related psychological distress measures, and reliability). The number of participants for Sample 1 and 2 satisfied standard psychometric sampling criteria for factor analysis, which generally suggest a ratio of roughly 10 respondents per item and a minimum sample size of 200 to 300 [57]. Parallel analysis with 200 random-data replications supplemented the Kaiser criterion. Five-category items were treated as approximately continuous; MLR was used as a sensitivity analysis to reduce the influence of non-normality. The hypothesized one-factor model and plausible alternative representations (a three-factor model and a bifactor model) were evaluated. Model diagnostics in Sample 2 indicated four localized residual associations corresponding to overlapping item content or wording. These four residual covariances were specified in a revised one-factor model and then held fixed, without further respecification, in the non-overlapping Sample 1 internal holdout. This procedure evaluates internal stability within the same convenience sample and does not constitute external replication. Gender invariance was evaluated sequentially. After full scalar invariance failed the |ΔCFI| ≤ 0.01 criterion, score tests were used to identify candidate intercepts for partial scalar invariance. Latent means were compared under the resulting partial scalar model, and partial strict invariance was tested as its direct continuation.

First, item analysis was performed using the critical ratio (CR) method and item–total correlations to assess the discriminatory power and homogeneity of the items.

Second, EFA was conducted using the Maximum Likelihood (ML) extraction method to explore the underlying factor structure of the SCS. The number of factors was determined based on eigenvalues and the scree plot.

Third, CFA was performed on Sample 2 to evaluate the hypothesized one-factor structure of the SCS. Model fit was evaluated using the ratio of chi-square to degrees of freedom (χ^2^/df), Root Mean Square Error of Approximation (RMSEA), Comparative Fit Index (CFI), Tucker–Lewis Index (TLI), and Standardized Root Mean Square Residual (SRMR). Values of χ^2^/df < 3, RMSEA < 0.08, SRMR < 0.08, and CFI/TLI > 0.90 were used as descriptive benchmarks [58] rather than mechanical decision rules. ML was retained for comparability with prior SCS studies and because the five-category items were treated as approximately continuous; MLR was used to examine robustness to non-normality. Candidate residual covariances were considered only when model diagnostics coincided with clear item-content or wording overlap, and the resulting specification was evaluated unchanged in the internal holdout. Pearson correlations with depression, anxiety, and stress were examined as hypothesis-based convergent validity evidence involving theoretically related variables.

## 3. Results

### 3.1. Item Analysis

Table 2 presents the item analysis results. The upper and lower 27% groups contained 88 and 103 participants, respectively, because tied scores clustered at the cut points. Critical ratio values ranged from 14.35 to 23.45 (all *p* < 0.001). Corrected item–total correlations, which exclude each item from its comparison total, ranged from 0.59 to 0.73. Cronbach’s alpha did not increase after deletion of any item (0.890-0.899); therefore, all 10 items were retained. The extreme group critical ratio method is reported for continuity with earlier SCS studies, while the corrected item–total correlations provide the more contemporary discrimination index.

### 3.2. EFA

The suitability of the data was assessed before conducting EFA. The Kaiser–Meyer–Olkin (KMO) measure was 0.923, and Bartlett’s test of sphericity was significant (χ^2^ = 1538.51, *p* < 0.001), indicating that the data were suitable for factor analysis. Under the prespecified one-factor extraction, the factor explained 48.83% of the variance; all 10 items loaded on it, with loadings from 0.63 to 0.77 and communalities from 0.40 to 0.59 (see Table 3). These findings were compatible with a substantial general factor, but the number of factors was evaluated further by parallel analysis and CFA.

Parallel analysis (factor-based, 200 random data replications) suggested three factors rather than the single factor indicated by the Kaiser criterion. In the exploratory three-factor solution, Items 7 and 8 had loadings of at least 0.30 on more than one factor, and the factor correlations were high (φ = 0.725–0.747); the solution was therefore not simple or clearly separable. The one-factor EFA is retained for direct comparison with prior SCS validations, but the parallel analysis result is reported transparently and evaluated further through the CFA model comparisons below.

### 3.3. CFA

CFA was initially conducted to evaluate the theoretical one-factor structure of the SCS. The original model fit poorly (χ^2^ = 180.91, df = 35, *p* < 0.001, χ^2^/df = 5.17, RMSEA = 0.114, CFI = 0.899, TLI = 0.871), indicating that a single latent factor did not fully account for the covariance among the items.

To evaluate sensitivity to non-normality, the initial one-factor model was re-estimated with MLR while treating the five-category items as approximately continuous. Fit remained poor (scaled χ^2^(35) = 134.83, robust CFI = 0.907, robust TLI = 0.880, robust RMSEA = 0.109), so the initial misfit was not explained by ordinary ML alone. The modified one-factor model was also re-estimated with MLR and showed robust CFI = 0.981, robust TLI = 0.972, robust RMSEA = 0.052, and SRMR = 0.033.

Inspection of model diagnostics indicated four areas of localized residual association: Items 9 and 10 (MI = 42.96), Items 8 and 9 (MI = 21.08), Items 1 and 10 (MI = 18.07), and Items 1 and 6 (MI = 17.54). Residual covariances can represent item-specific dependence beyond the common factor when paired items share wording or content [59,60].

Accordingly, four residual covariances were specified to represent plausible local dependence. Items 8 and 9 both concern highly sensitive secrets; Items 9 and 10 concern negatively valenced internal content that is withheld; Items 1 and 10 share closely parallel wording about information not shared with anyone; and Items 1 and 6 both concern maintaining secrecy and preventing disclosure. These terms were not part of the original model; the revised specification was therefore held fixed and evaluated in Sample 1 without additional residual paths.

The revised one-factor model yielded χ^2^ = 68.87, df = 31, *p* < 0.001, χ^2^/df = 2.22, RMSEA = 0.062 (90% CI: 0.042–0.081), CFI = 0.974, TLI = 0.962, and SRMR = 0.033. These indices indicate that a one-factor representation including four local dependence terms described Sample 2 adequately; they do not establish strict unidimensionality. Standardized factor loadings ranged from 0.63 to 0.77 (all *p* < 0.001). Average Variance Extracted (AVE) was 0.465 and Composite Reliability (CR) was 0.896. The AVE was below the conventional 0.50 threshold and therefore indicates marginal item-level convergence despite acceptable composite reliability [61].

The revised specification was applied unchanged to Sample 1 as a non-overlapping internal holdout check. Fit was acceptable but weaker than in Sample 2 (ML: χ^2^(31) = 91.89, CFI = 0.960, TLI = 0.942, RMSEA = 0.078, SRMR = 0.039; MLR: scaled χ^2^(31) = 68.10, robust CFI = 0.967, TLI = 0.952, robust RMSEA = 0.071, SRMR = 0.039). This result supports internal stability of the specified residual pattern but does not constitute external replication. A data-informed three-factor CFA fit worse than the revised one-factor model (χ^2^(32) = 131.15, CFI = 0.932, TLI = 0.904, RMSEA = 0.098). The bifactor model produced an improper Heywood solution, including a negative residual variance and unavailable standard errors, so its fit indices were not interpreted. The two-factor model reported by Costa et al. [35] was not imposed because that version added two health-related items that are absent from the original 10-item SCS used here. Overall, the revised one-factor representation provided the most parsimonious account among the models examined; the remaining dimensional ambiguity requires independent investigation.

### 3.4. Convergent Validity

As shown in Table 4, SCS total scores were moderately and positively correlated with depression (r = 0.35), anxiety (r = 0.37), and stress (r = 0.35), all *p* < 0.01. This pattern was consistent with the hypothesized relation between self-concealment and psychological distress.

### 3.5. Reliability

Internal consistency was high: Cronbach’s α was 0.896, and split-half reliability (maximum Spearman–Brown coefficient) was 0.933. As a supplementary internal consistency estimate, McDonald’s omega was also computed (ω = 0.897), corroborating the Cronbach’s alpha result.

### 3.6. Gender Invariance

Configural (M1), metric/weak (M2), full scalar/strong (M3), partial scalar (M3p), and partial strict (M4p) models were examined across women (N = 429) and men (N = 215). Model fit and the prespecified |ΔCFI| ≤ 0.01 decision rule are reported in Table 5.

Metric invariance was supported (M2 vs. M1: ΔCFI = 0.0004; ΔRMSEA = −0.0053). Full scalar invariance was not supported because M3 reduced CFI by 0.0150 relative to M2. A score test identified the Item 1 intercept as the largest source of non-invariance (χ^2^ = 26.59, *p* < 0.001). Freeing this intercept while constraining the other nine item intercepts produced partial scalar invariance (M3p vs. M2: ΔCFI = −0.0062; ΔRMSEA = 0.0017). When residual variances were then constrained in the partial strict model, ΔCFI was −0.0103 and ΔRMSEA was 0.0034 relative to M3p. Because the absolute ΔCFI slightly exceeded 0.01, partial strict invariance was borderline and was not supported under the prespecified rule. Thus, the data support configural, metric, and partial scalar invariance, but not full scalar or partial strict invariance.

Gender differences were evaluated primarily through a latent-mean likelihood-ratio test under the partial scalar model. Constraining the latent means to equality did not worsen fit, Δχ^2^(1) = 0.066, *p* = 0.798, providing no evidence of a gender difference in latent self-concealment. As a descriptive supplement, observed total scores also did not differ significantly between women (M = 28.33) and men (M = 27.94), Welch t = 0.48, *p* = 0.63. The latent-mean test is preferred because Item 1 showed intercept non-invariance.

## 4. Discussion

The Persian SCS exhibited strong internal consistency. Medium positive correlations were observed between the self-concealment and depressive, anxiety, and stress symptoms, which demonstrates moderate convergent validity of the Persian SCS. The initial single-factor measurement model yielded unsatisfactory fit indices; specification of four localized residual correlations between paired items substantially improved model performance. The revised unidimensional structure achieved adequate model fit in Sample 2, with acceptable fit retained in the internal holdout subsample. The AVE fell below the conventional threshold of 0.50, and the measurement invariance analysis only supported partial scalar invariance across gender groups. Taken together, the findings provide preliminary evidence for research use of the Persian SCS while indicating the need for independent replication.

The dimensionality evidence was mixed. The one-factor extraction was comparable with earlier SCS studies [5,27,32,33,37,38,39,40,41], whereas parallel analysis suggested three factors. However, the exploratory factors were strongly correlated, and the three-factor CFA fit worse than the revised one-factor model. Together, these findings are consistent with a substantial general factor accompanied by local item dependence; they do not demonstrate strict unidimensionality. A two-factor latent structure was identified in an extended SCS scale supplemented with two extra health-focused items [35]. As these supplementary indicators were not included in the current 10-item instrument, direct replication of this published factor structure was unfeasible, as doing so would require arbitrary and empirically unsubstantiated item assignments. More generally, prior work has adopted item addition and elimination strategies [35,37], highlighting persistent ambiguity surrounding the measure’s latent structure. The present findings should therefore be interpreted as evidence about the original 10-item Persian SCS rather than as a final resolution of the dimensionality debate.

The first extracted factor accounted for 48.83% of the total variance, within the 33.37–55.66% range reported in previous SCS studies [33,34,39,40]. This comparison does not by itself establish unidimensionality.

Alternative models did not provide a clearer account of the data. The three-factor CFA fit worse than the revised one-factor model, and the bifactor model produced an improper solution with a negative residual variance and unavailable standard errors. Among the models examined, the revised one-factor model therefore provides the most parsimonious representation, while the underlying dimensionality remains open to independent study.

Modest correlations with PHQ-2, GAD-2, and PSS-4 scores (r = 0.35–0.37) offered partial convergent validity support, which replicated previous findings [6]. However, many other critical psychometric properties are still needed, such as test–retest reliability, discriminant, known-groups, and incremental validity.

The gender invariance analysis supported metric and partial scalar invariance after the Item 1 intercept was freed. The latent-mean test under this partial scalar model found no evidence of a gender difference. Partial strict invariance was borderline and did not meet the prespecified |ΔCFI| ≤ 0.01 rule. The unequal group sizes and Item 1 non-invariance warrant caution when interpreting observed-score comparisons.

The full scalar model failed the prespecified ΔCFI criterion, whereas the partial scalar model met it after freeing the Item 1 intercept. The corresponding latent-mean likelihood-ratio test was non-significant, Δχ^2^(1) = 0.066, *p* = 0.798. This conclusion is more defensible than relying on the observed-score t test alone, but it remains conditional on partial rather than full scalar invariance, which mirrors previous research findings [37].

Gender differences were interpreted primarily using the latent-mean test under the partial scalar model; the observed-score t test was retained as a descriptive supplement. Neither analysis provided evidence of a gender difference in self-concealment in the present sample. Previous studies have reported mixed gender findings [32,34,38,58]. Differences across studies may reflect sampling, age, cultural context, or the types of information concealed, and these possibilities should be examined directly in future research.

Several limitations should be considered. First, the online convenience sample may not represent the broader Iranian adult population, and adults with limited internet access or digital literacy may be underrepresented. The unequal gender group sizes further limit generalizability. Second, the cross-sectional self-report design did not permit evaluation of test–retest reliability, longitudinal stability, measurement error over time, discriminant validity, or known groups validity.

Third, the original one-factor model did not fit well. The four localized residual associations in the revised model were identified during model diagnostics and corresponded to item content or wording overlap; their unchanged performance in the internal holdout is informative but requires replication in an independent sample. Fourth, AVE was below 0.50, and full scalar and partial strict gender invariance were not supported. These points limit claims of strict unidimensionality, broad validity, and unrestricted group comparisons. Finally, the pilot test of the pre-final Persian version was only administrated in eight participants, which might be insufficient for a large-scale validation study and a larger pilot group would have provided more robust feedback on the semantic clarity of the Persian items.

## 5. Conclusions

In conclusion, the Persian SCS showed high internal consistency, moderate associations with psychological distress indicators, and metric and partial scalar invariance across gender. Structural evidence was qualified: the original one-factor model fit poorly, the revised model accounted for four localized item pair residual associations, parallel analysis suggested three factors, and AVE was below 0.50. The scale may be used cautiously for research in similar Iranian adult samples, but it should not yet be regarded as fully validated for clinical screening or unrestricted gender comparisons. Independent replication, longitudinal reliability, and broader validity testing are required.

## Figures and Tables

**Table 1:** The demographic characteristics of the 644 participants.

Demographic Dimension	Number	Percentage
Gender		
Female	429	66.6%
Male	215	33.4%
Age		
18–20	103	16.0%
21–30	243	37.7%
31–40	174	27.0%
41–50	93	14.4%
51–60	26	4.0%
60+	5	0.8%
Educational attainment		
≤Elementary school	13	2.0%
Middle school	30	4.7%
High school	169	26.2%
Postgraduate	50	7.8%
Bachelor	225	34.9%
Master	129	20.0%
Phd	28	4.3%
Marital status		
Single	296	46.0%
Married	319	49.5%
Divorced/widowed	27	4.2%
Employment status		
Unemployed	94	14.6%
Studying	155	24.1%
Full-time job	150	23.3%
Part-time job	95	14.8%
Freelancer	37	5.7%
Retired	26	4.0%
Other	87	13.5%

**Table 2:** The results of the item analysis of the Self-Concealment Scale.

Item	All Samples (N = 322)		Low Subgroup (N = 103)		High Subgroup (N = 88)		Critical Ratio	Corrected Item–Total Correlation	Cronbach’s α If Item Deleted
	Mean	SD	Mean	SD	Mean	SD			
1	3.13	1.45	1.86	1.18	4.28	0.87	16.27 ***	0.59 ***	0.899
2	2.54	1.24	1.52	0.64	3.69	1.15	15.75 ***	0.68 ***	0.893
3	2.99	1.36	1.78	0.93	4.20	0.85	18.90 ***	0.70 ***	0.891
4	2.75	1.30	1.65	0.75	4.05	0.96	19.00 ***	0.73 ***	0.890
5	2.83	1.28	1.87	1.00	3.93	0.98	14.35 ***	0.60 ***	0.897
6	2.68	1.26	1.80	0.88	3.86	1.05	14.59 ***	0.62 ***	0.897
7	3.04	1.26	1.97	1.00	4.15	0.74	17.24 ***	0.66 ***	0.894
8	2.88	1.33	1.62	0.78	4.24	0.76	23.45 ***	0.73 ***	0.890
9	2.23	1.2	1.37	0.67	3.28	1.29	12.58 ***	0.64 ***	0.895
10	2.57	1.27	1.61	0.74	3.75	1.17	14.81 ***	0.65 ***	0.895

Note. SD: Standard Deviation; *** *p* < 0.001.

**Table 3:** The results of the exploratory factor analysis of the Self-Concealment Scale (N = 322).

Item	Factor Loading	Communality
1	0.63	0.40
2	0.71	0.51
3	0.74	0.54
4	0.77	0.59
5	0.63	0.40
6	0.65	0.43
7	0.69	0.47
8	0.77	0.59
9	0.69	0.47
10	0.69	0.48

**Table 4:** Correlations of self-concealment scores with psychological distress indicators.

Variable	Self-Concealment	Depression	Anxiety	Stress
Self-concealment	1			
Depression	0.35 **	1		
Anxiety	0.37 **	0.69 **	1	
Stress	0.35 **	0.45 **	0.48 **	1
Mean	28.76	2.45	2.34	7.05
SD	9.16	1.83	1.87	2.87

Note. ** *p* < 0.01.

**Table 5:** The results of gender invariance of the Self-Concealment Scale.

Model	χ^2^	df	χ^2^/df	CFI	TLI	RMSEA	ΔCFI	ΔRMSEA
M1 Configural	177.74	62	2.87	0.962	0.945	0.076	-	-
M2 Metric/Weak	185.61	71	2.61	0.962	0.952	0.071	0.0004	−0.0053
M3 Full scalar	240.29	80	3.00	0.947	0.941	0.079	−0.0150	0.0081
M3p Partial scalar	212.61	79	2.69	0.956	0.950	0.072	−0.0062	0.0017
M4p Partial strict	253.93	89	2.85	0.946	0.945	0.076	−0.0103	0.0034

## Data Availability

The data presented in this study are available on request from the corresponding author due to ethical reasons.

## References

[1] Bianchi V., Greenaway K.H., Moeck E.K., Slepian M.L., Kalokerinos E.K. (2025). Secrecy in everyday life. Personal. Soc. Psychol. Bull..

[2] Bedrov A., Gable S.L. (2025). Secrets lurking in the background: Investigating the underlying effects of secrets in everyday life. J. Exp. Soc. Psychol..

[3] Slepian M.L. (2024). The new psychology of secrecy. Curr. Dir. Psychol. Sci..

[4] Le Forestier J.M., Lewis N.A. (2024). When and why people conceal their identities. Nat. Rev. Psychol..

[5] Larson D.G., Chastain R.L. (1990). Self-concealment: Conceptualization, measurement, and health implications. J. Soc. Clin. Psychol..

[6] Larson D.G., Chastain R.L., Hoyt W.T., Ayzenberg R. (2015). Self-concealment: Integrative review and working model. J. Soc. Clin. Psychol..

[7] Bedrov A., Gable S.L. (2024). Just between us…: The role of sharing and receiving secrets in friendship across time. Pers. Relatsh..

[8] Davis C.G., Tabri N. (2023). The secrets that you keep: Secrets and relationship quality. Pers. Relatsh..

[9] Frijns T., Finkenauer C. (2009). Longitudinal associations between keeping a secret and psychosocial adjustment in adolescence. Int. J. Behav. Dev..

[10] Salinas-Oñate N., Flores M.J.S., Salazar-Fernández C., Baeza-Rivera M.J., Olivera M. (2024). Self-concealment, depressive symptoms, and seeking professional help: Evidence of an invariance model. Behav. Psychol./Psicol. Conduct..

[11] Leleux-Labarge K., Hatton A.T., Goodnight B.L., Masuda A. (2015). Psychological Distress in Sexual Minorities: Examining the Roles of Self-Concealment and Psychological Inflexibility. J. Gay Lesbian Ment. Health.

[12] Dennis M.T., Davis C.G. (2026). Out of Sight but on My Mind: Distal Stressors, Identity Concealment, Proximal Stress, and Mental Health Among Sexual or Gender Minorities. Sex. Res. Soc. Policy.

[13] Khan Z.K., Raghavan C. (2025). Self-Concealment as a Trauma Response: Examining the Mediating Role of Shame and Dissociation in the Link Between PTSD Symptoms and Self-Concealment Among Women Who Were Sex-Trafficked. J. Hum. Traffick..

[14] Liu F., Wang N., Wang Y., Ye Z., Jian C. (2024). The hidden influence: How self-concealment shapes the effect of trauma centrality on PTSD symptoms and posttraumatic growth. Curr. Psychol..

[15] Chung M.C., Chen Z.S., Han B.X. (2022). The impact of anger and self-concealment on post-traumatic stress and psychiatric comorbid symptoms in Chinese prisoners: A longitudinal study. Crim. Behav. Ment. Health.

[16] Chen X., Ma Q., Peng X., Yang H., Ye Z., Yang C., He C. (2023). Mediating effect of self-concealment between non-suicidal self-injury and internet addiction in college students: A cross-sectional study. BMC Psychol..

[17] Burke T.A., Ammerman B.A., Hamilton J.L., Stange J.P., Piccirillo M. (2020). Nonsuicidal self-injury scar concealment from the self and others. J. Psychiatr. Res..

[18] Raghav K.S., Devi M., Mahadevaswamy M., Nathawat S. (2025). Influence of smartphone addiction on suicidal ideation and nonsuicidal self-injury among college students: The mediating role of self-concealment. Arch. Ment. Health.

[19] Hogge I., Kim J., Kim E. (2023). The Burden of Keeping Things to Yourself: Self-Concealment and Suicidality. Couns. Psychol. Q..

[20] Friedlander A., Nazem S., Fiske A., Nadorff M.R., Smith M.D. (2012). Self-Concealment and Suicidal Behaviors. Suicide Life-Threat. Behav..

[21] Hogge I., Blankenship P. (2020). Self-concealment and suicidality: Mediating roles of unmet interpersonal needs and attitudes toward help-seeking. J. Clin. Psychol..

[22] D’Agata M.T., Granek J.A., Holden R.R., Nazarov A. (2021). The Relation between Self-Concealment and Self-Reported Mental Health Symptoms in a Sample of Canadian Armed Forces Personnel. Mil. Behav. Health.

[23] Hartman J.D., Patock-Peckham J.A., Corbin W.R., Gates J.R., Leeman R.F., Luk J.W., King K.M. (2015). Direct and indirect links between parenting styles, self-concealment (secrets), impaired control over drinking and alcohol-related outcomes. Addctv. Behav..

[24] Chinweuba D.C., Ifeagwazi C.M., Chinweuba A.U., Chukwuorji J.C. (2023). Does self-concealment and self-compassion differentially influence substance use for male versus female adolescents?. J. Subst. Use.

[25] Jo D., Hill M.L., Masuda A. (2025). Difficulty in Emotion Regulation and Self-Concealment as Mediators of the Link Between Psychological Distress and Disordered Eating Behavior in Emerging Adult Women. Behav. Sci..

[26] Masuda A., Latner J.D., Barlie J.P., Sargent K. (2018). Understanding self-concealment within a framework of eating disorder cognitions and body image flexibility: Conceptual and applied implications. Eat. Behav..

[27] González-Menéndez A., González-Suárez V., Medrano M.J., Alvarez M.-J., Postigo Á. (2025). Psychometric Properties of the Self-Concealment Scale in Spanish Adolescents: Adaptation and Validation for Eating Disorder Risk Assessment. Actas Esp. Psiquiatr..

[28] Zhou G., Wang E. (2021). Effects of self-concealment and self-esteem on Internet addiction in college students. Soc. Behav. Personal..

[29] Yayla İ.E., Makas S., Koçak L., Yıldırım M., Dombak K. (2025). Moderated Mediation in Problematic Digital Gaming Among Adolescents: Self-Esteem, Social Anxiety, Self-Concealment, and Sensation Seeking. Children.

[30] Chang L., Xu J., Zhang L., Yin Y., Zhang H. (2025). Linking Self-Concealment to Problematic Short Video Use: Online Social Support and Fear of Missing out as a Serial Mediator. J. Genet. Psychol..

[31] Ritz T., Dahme B. (1996). Repression, self-concealment and rationality/emotional defensiveness: The correspondence between three questionnaire measures of defensive coping. Personal. Individ. Differ..

[32] Kawano K. (2001). Correlational Analysis among Japanese Self-Concealment Scale, Kida’s Stimulus-Seeking Scale and self-reported physical symptoms. Jpn. J. Exp. Soc. Psychol..

[33] Wang C.K. (2002). The factor structure of the Chinese adaptation of self-concealment scale in middle school students. Chin. J. Appl. Psychol..

[34] Deniz M., Çok F. (2010). Psychometric properties and adaptation of the Self-Concealment Scale to the Turkish adolescents. Elem. Educ. Online.

[35] Costa M., Pereira A.T., Soares M.J., Azevedo J., Macedo A. (2016). Self-Concealment Scale: Validation of two Portuguese versions. Eur. Psychiatry.

[36] Letelier C., Errázuriz P. (2020). Adaptación y Validación en Español de la Self-Concealment Scale. Psykhe.

[37] Salinas-Onate N., Baeza-Rivera M.J., Salinas-Rehbein B., Escandon-Nagel N., Escobar-Alaniz B. (2022). Validation of the Adapted Version of the Larson & Chastain Self-Concealment Scale in Chilean University Students/Validación de la Versión Adaptada de la Escala de Auto-Ocultamiento de Larson & Chastain en Universitarios Chilenos. Rev. Iberoam. Diagnóstico Y Eval.-E Aval. Psicol..

[38] Cramer K.M., Barry J.E. (1999). Psychometric properties and confirmatory factor analysis of the self-concealment scale. Personal. Individ. Differ..

[39] Fan Z., Shi X., Huang X., Li L. (2024). Self-concealment scale (SCS) in middle-aged people: Psychometric features and cross-age equivalence test. BMC Psychiatry.

[40] Fan Z., Shi X., Hu C., Zhu L., Wang Z. (2023). Reliability and Validity of Self-Concealment Scale in Chinese Older Adults. Psychol. Res. Behav. Manag..

[41] Wismeijer A.A.J., Sijtsma K., van Assen M.A.L.M., Vingerhoets A.J.J.M. (2008). A Comparative Study of the Dimensionality of the Self-Concealment Scale Using Principal Components Analysis and Mokken Scale Analysis. J. Personal. Assess..

[42] Leitgöb H., Seddig D., Asparouhov T., Behr D., Davidov E., De Roover K., Jak S., Meitinger K., Menold N., Muthén B. (2023). Measurement invariance in the social sciences: Historical development, methodological challenges, state of the art, and future perspectives. Soc. Sci. Res..

[43] Davoodi E., King K., Singh A., Jobson L. (2025). Exploring the effect of gender and culture on the relationship between self-silencing and depressive symptoms. Aust. Psychol..

[44] Arjmand R., Ziari M. (2020). Sexuality and concealment among Iranian young women. Sexualities.

[45] Dustin M. (2026). Putting People in Boxes: Iranian Exiles and Queer Identities. Sex. Cult..

[46] Taghva A., Hajebi A., Noorbala A.A., Khademi M., Asadi A., Rahnejat A.M., Alizadeh K., Shalbafan M., Bolhari J., Hashemian S.S. (2025). Mental health stigma in Iran: A systematic review. Stigma Health.

[47] Taghva A., Farsi Z., Javanmard Y., Atashi A., Hajebi A., Khademi M. (2017). Stigma barriers of mental health in Iran: A qualitative study by stakeholders of mental health. Iran. J. Psychiatry.

[48] Eskandari L., Keramat A., Baghini G.S., Fardid M., Rohani-Rasaf M. (2025). Iranian Women’s Experiences of Unintended Pregnancy and Induced Abortion: A Meta-Synthesis Study. Iran. J. Med. Sci..

[49] Kroenke K., Spitzer R.L., Williams J.B. (2003). The Patient Health Questionnaire-2: Validity of a two-item depression screener. Med. Care.

[50] Levis B., Sun Y., He C., Wu Y., Krishnan A., Bhandari P.M., Neupane D., Imran M., Brehaut E., Negeri Z. (2020). Accuracy of the PHQ-2 Alone and in Combination With the PHQ-9 for Screening to Detect Major Depression: Systematic Review and Meta-analysis. JAMA.

[51] Ghazisaeedi M., Mahmoodi H., Arpaci I., Mehrdar S., Barzegari S. (2022). Validity, Reliability, and Optimal Cut-off Scores of the WHO-5, PHQ-9, and PHQ-2 to Screen Depression Among University Students in Iran. Int. J. Ment. Health Addict..

[52] Kroenke K., Spitzer R.L., Williams J.B.W., Monahan P.O., Löwe B. (2007). Anxiety Disorders in Primary Care: Prevalence, Impairment, Comorbidity, and Detection. Ann. Intern. Med..

[53] Aktürk Z., Hapfelmeier A., Fomenko A., Dümmler D., Eck S., Olm M., Gehrmann J., von Schrottenberg V., Rehder R., Dawson S. (2025). Generalized Anxiety Disorder 7-item (GAD-7) and 2-item (GAD-2) scales for detecting anxiety disorders in adults. Cochrane Database Syst. Rev..

[54] Veisy F., Farahani H., Togha M., Gharaee B., Janani L., Aghebati A. (2021). Rapid screening for generalized anxiety disorder in patients with migraine. Curr. J. Neurol..

[55] Warttig S.L., Forshaw M.J., South J., White A.K. (2013). New, normative, English-sample data for the Short Form Perceived Stress Scale (PSS-4). J. Health Psychol..

[56] Maroufizadeh S., Zareiyan A., Sigari N. (2014). Psychometric properties of the 14, 10 and 4-item “Perceived Stress Scale” among asthmatic patients in Iran. Payesh.

[57] Clark L.A., Watson D. (2019). Constructing validity: New developments in creating objective measuring instruments. Psychol. Assess..

[58] Hu L.t., Bentler P.M. (1999). Cutoff criteria for fit indexes in covariance structure analysis: Conventional criteria versus new alternatives. Struct. Equ. Model..

[59] Kline P. (2015). A Handbook of Test Construction (Psychology Revivals): Introduction to Psychometric Design.

[60] Byrne B.M. (2013). Structural Equation Modeling with Mplus: Basic Concepts, Applications, and Programming.

[61] Fornell C., Larcker D.F. (1981). Evaluating Structural Equation Models with Unobservable Variables and Measurement Error. J. Mark. Res..

